# Structural basis of Keap1 interactions with Nrf2

**DOI:** 10.1016/j.freeradbiomed.2015.05.034

**Published:** 2015-11

**Authors:** Peter Canning, Fiona J. Sorrell, Alex N. Bullock

**Affiliations:** Structural Genomics Consortium, University of Oxford, Old Road Campus, Oxford OX3 7DQ, UK

**Keywords:** Keap1, Nrf2, BTB, Kelch, Cullin, Ubiquitin, Free radicals

## Abstract

Keap1 is a highly redox-sensitive member of the BTB-Kelch family that assembles with the Cul3 protein to form a Cullin–RING E3 ligase complex for the degradation of Nrf2. Oxidative stress disables Keap1, allowing Nrf2 protein levels to accumulate for the transactivation of critical stress response genes. Consequently, the Keap1–Nrf2 system is extensively pursued for the development of protein–protein interaction inhibitors that will stabilize Nrf2 for therapeutic effect in conditions of neurodegeneration, inflammation, and cancer. Here we review current progress toward the structure determination of Keap1 and its protein complexes with Cul3, Nrf2 substrate, and small-molecule antagonists. Together the available structures establish a rational three-dimensional model to explain the two-site binding of Nrf2 as well as its efficient ubiquitination.

## Highlights

• Keap1 and Nrf2 are multidomain proteins that determine the antioxidant response.
• Structural models suggest how Keap1 assembles into a Cullin–RING ligase.
• Dimeric Keap1 supports a two-site tethering mechanism for Nrf2 ubiquitination.
• Crystal structures reveal the mechanism of action of chemical inhibitors of Keap1.

## Graphical abstract

**Figure:**
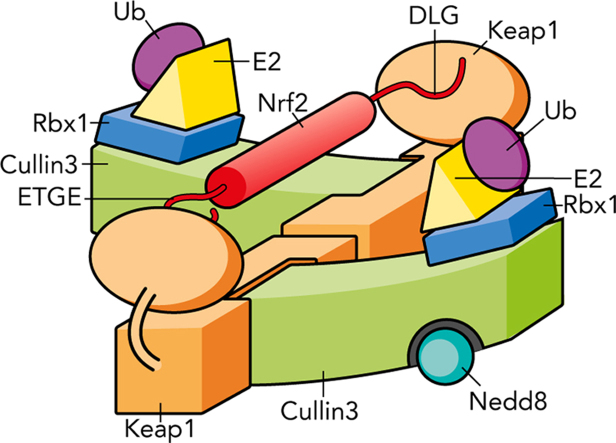

## 1 Introduction

Nrf2, encoded by the gene *NFE2L2*[1], belongs to the Cap’n’Collar (CNC) subfamily of basic leucine zipper (bZIP) transcription factors, which in vertebrates comprises NFE2 (nuclear factor erythroid-derived 2) and the NFE2-related factors Nrf1, Nrf2, and Nrf3 [2]. Nrf2 has become the best known family member for its cytoprotective role in the responses to oxidative and electrophilic stress [3], [4], [5]. This activity is dependent on its transactivation function and its dimerization with members of the small Maf (musculoaponeurotic fibrosarcoma oncogene homolog) protein family [6]. The Nrf2–Maf heterodimer is targeted specifically to genes containing antioxidant-response elements in their regulatory regions, including many antioxidant and detoxification enzymes, ABC transporters, and other stress response proteins [7], [8].

Under normal conditions, the levels of Nrf2 protein are kept low by the E3 ubiquitin ligase Keap1 (Kelch ECH-associating protein 1), which ubiquitinates Nrf2 in the cytoplasm and targets it for degradation by the 26 S proteasome [9], [10], [11], [12], [13], [14], [15]. This constitutive degradation of Nrf2 allows for only the basal expression of its target stress response genes as part of a housekeeping function. However, under conditions of oxidative stress, or in the presence of electrophilic xenobiotics, the activity of Keap1 is diminished and Nrf2 can accumulate in the nucleus where it activates the inducible high expression of its target genes. In this manner, Keap1 functions as a critical sensor of cellular stress. Its high redox sensitivity is determined by a number of cysteine residues that are distributed throughout the Keap1 protein and are vulnerable to oxidation or to covalent modification by electrophiles. Notably, different reactive chemicals appear to target different cysteines in Keap1, giving rise to the concept of a “cysteine code” [4], [16], [17]. Two residues, Cys273 and Cys288, seem essential for Keap1 to control Nrf2 under both basal and stress conditions, whereas Cys151 is primarily required under conditions of stress [18], [19], [20], [21]. Other Keap1 residues, including Cys226, Cys434, and Cys613, seem important for sensing specific toxins.

As a master regulator of the antioxidant response, the Keap1–Nrf2 system protects cellular proteins and DNA from oxidative damage caused by reactive oxygen species and electrophiles. In addition, it controls transporters critical for cellular detoxification. Consequently, the Keap1–Nrf2 system has emerged as an important therapeutic target in cancer and neurodegenerative conditions, as well as many autoimmune and inflammatory diseases [2], [4], [5], [16], [22]. Critical for such intervention strategies is a deep understanding of the structure and function of these proteins. Progress in this respect has relied on the definition of the domain architecture of the Keap1 and Nrf2 proteins and the identification of the specific regions mediating their interactions [23].

**Fig. 1:**
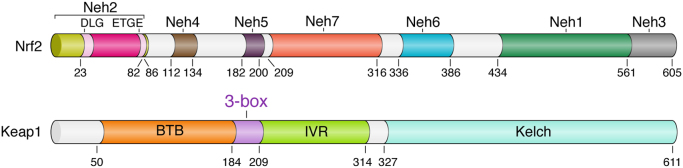
Domain architecture of the Keap1 and Nrf2 proteins. Domain boundaries and residue numbers are shown for the human proteins.

The Nrf2 protein in humans is 605 amino acids long and contains seven highly conserved regions known as Nrf2-ECH homology (Neh) domains (Fig. 1). Neh1 contains the CNC–bZIP domain, which mediates heterodimerization with Maf [6]. The Neh2 domain contains the two degrons that are specifically bound by Keap1. These are commonly known as the DLG and ETGE motifs after their sequence conservation in the single-letter amino acid code [24]. Two further redox-independent degrons have since been described in Neh6 [25]. These motifs are not recognized by Keap1, but are instead targeted for degradation by the E3 ubiquitin ligase β-TrCP. This alternative pathway is enhanced by the phosphorylation of Nrf2 by glycogen synthase kinase-3β, providing another mechanism for cellular control of Nrf2 activities [26], [27], [28]. The Neh3–5 domains are thought to function in transactivation by binding to various components of the transcriptional apparatus [29], [30]. Conversely, repression of Nrf2 is conferred by the interactions of Neh7 with the DNA-binding domain of retinoic X receptor α [31].

Keap1 belongs to the BTB-Kelch family of proteins, which comprises some 50 members variously named as Kelch-like 1–42 (KLHL1–42) or Kelch and BTB domain-containing 1–14 (KBTBD1–14) [32], [33]. All of these proteins assemble with Cullin 3 (herein Cul3) and Rbx1 to form multisubunit Cullin–RING (really interesting new gene) ligases (CRLs) for protein ubiquitination. Keap1 is classified as KLHL19 and comprises three domains spanning some 611 amino acids (Fig. 1). The N-terminal BTB domain is named after the *Drosophila* proteins Broad complex, Tramtrack, and Bric à brac, in which it was first identified [34]. The BTB domain mediates the homodimerization of Keap1 and contributes additionally to its interaction with Cul3 [35]. A further Cul3 interaction is provided by the 3-box motif, which forms the proximal part of the central intervening region (IVR domain) [32]. The C-terminal Kelch domain is required for substrate capture and can bind separately to the ETGE [36], [37] or DLG [38], [39] motifs of Nrf2.

To date there are no available high-resolution structures describing either of the full-length Keap1 or Nrf2 proteins. Nonetheless, a number of crystal structures featuring Keap1, or its BTB-Kelch family homologs, have revealed the molecular mechanisms determining its interactions with Nrf2 substrate or Cul3 protein, as well as the action of chemical inhibitors that stabilize Nrf2 for therapeutic gain.

## 2 Structural basis of Nrf2 binding to the Kelch domain of Keap1

Nuclear magnetic resonance spectroscopy has shown the Neh2 region of Nrf2 to be intrinsically disordered [24], but capable of binding to the full-length Keap1 protein at low nanomolar concentrations (*K*_D_ value ~5 nM) [24], [40]. This binding was replicated by a 16-residue peptide (AFFAQLQLDE*ETGE*FL) incorporating amino acids 69–84 of Nrf2, which flank the conserved ETGE motif [24]. Subsequently, the molecular nature of this interaction was captured by two high-resolution crystal structures. The structure of the same 16-residue Nrf2 peptide was solved at 1.5- Å resolution in complex with the Kelch domain of human Keap1 [36]. A further structure was solved independently at 1.7- Å resolution comprising the equivalent mouse Kelch domain and a shorter peptide spanning residues 76–84 of Nrf2 [37]. Additionally, crystal structures have been reported for the human and mouse Kelch domains in the absence of ligand [37], [41], [42].

Overall, the Kelch domain contains six Kelch repeats that fold into a six-bladed β-propeller structure [42]. Each blade (I–VI) comprises a four-stranded antiparallel β-sheet (β strands A–D), in which the shorter βA strands form the central core. The final βA strand from the C-terminal region (CTR) closes the propeller by completing blade I. The Kelch repeats are notably diverse in sequence, allowing for substrate selectivity, but contain a limited number of conserved positions that maintain the overall fold [32], [43]. These include a double-glycine repeat (DGR) that terminates the βB strand as well as individual tyrosine (βC) and tryptophan (βD) residues that mediate hydrophobic packing between blades. Based on this consensus, the Kelch domain has also been described as the DGR or DC (DGR and CTR) domain [37], [43], [44].

**Fig. 2:**
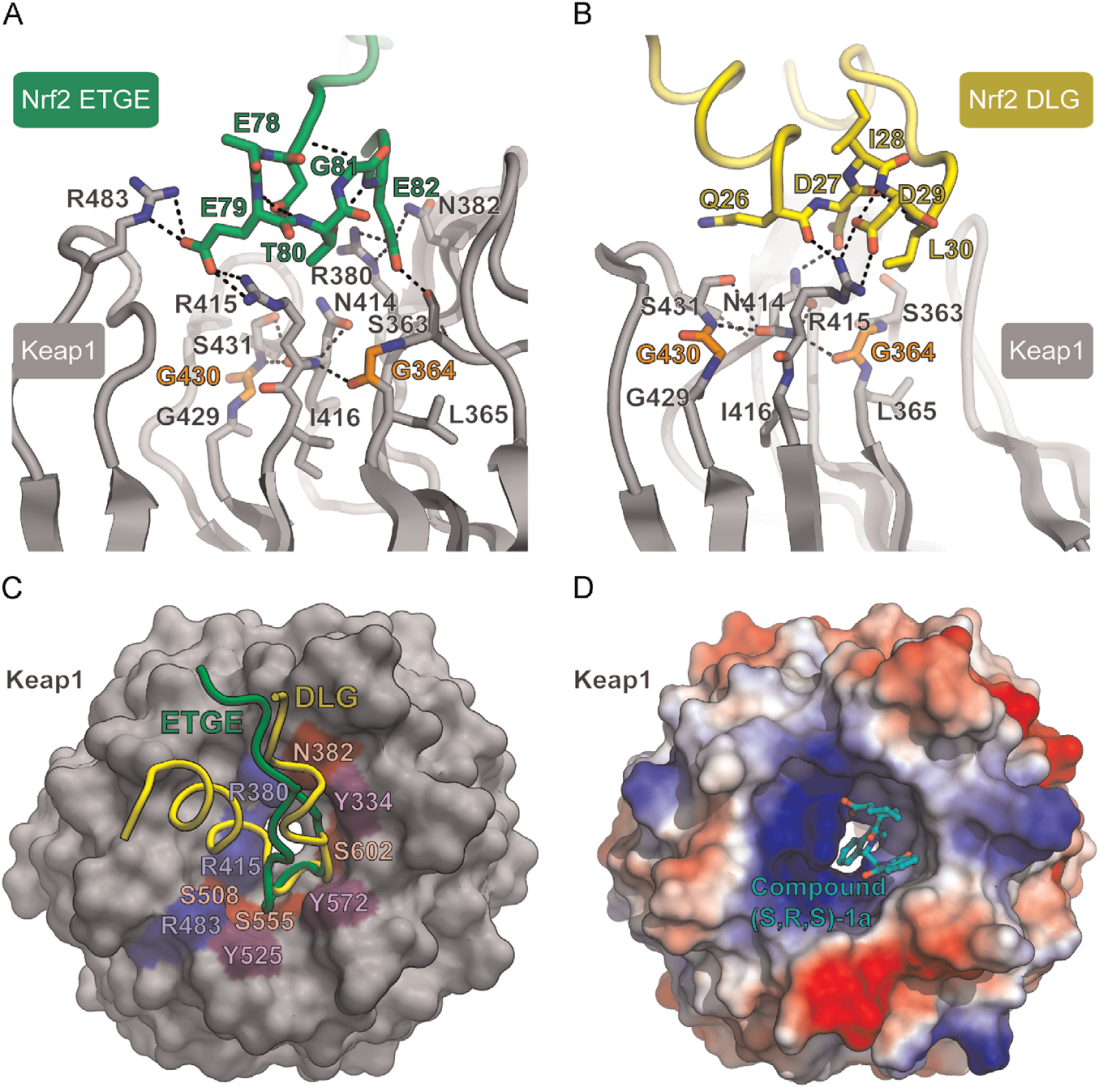
Binding of Nrf2 to Keap1. (A) Selected side-chain interactions are shown in the complex of human Keap1 and the Nrf2 ETGE motif (PDB 2FLU). Kelch domain positions with known somatic cancer mutations (G364C and G430C) are shown in orange; other Keap1 and Nrf2 interface residues are shown in gray and green, respectively. (B) Selected side-chain interactions in the DLG motif complex with mouse Keap1 (PDB 3WN7). DLG peptide residues are colored yellow; Keap1 residues are colored as in (A). (C) Comparison of the binding of the ETGE (green) and DLG (yellow) peptides. Colored areas on the Keap1 surface indicate the main interacting residues (blue, basic; red, polar; purple, hydrophobic). (D) Structural basis for Keap1 inhibition by small molecules targeting the Kelch domain. The electrostatic potential of the protein surface reveals a basic patch around the Nrf2 binding site. A bound small-molecule inhibitor is shown from PDB 4L7B (chain B) [70].

The substrate binding surface lies on one face of the Kelch domain, where a shallow pocket is created by the long loops that connect β-strands D and A (DA loop) as well as β-strands B and C (BC loop). The bound ETGE peptide of Nrf2 adopts a β-turn conformation that inserts into this pocket to establish a buried surface area of 420 Å^2^ (Fig. 2A) [36], [37]. Specific electrostatic interactions are made by both glutamate residues in the ETGE motif. Glu79 in Nrf2 forms hydrogen bonds with Keap1 residues Arg415, Arg483, and Ser508, whereas Glu82 hydrogen bonds with Keap1 residues Ser363, Asn382, and Arg380. Further electrostatic contacts mediated through water or the peptide backbone are supplemented by additional van der Waals interactions.

The conserved DLG motif in the Neh2 region of Nrf2 was identified as a second independent binding site with 100-fold weaker affinity for Keap1 [24]. Its complex with the Kelch domain of mouse Keap1 was solved initially at 1.9- Å resolution using a peptide spanning amino acids 22–36 of Nrf2 [38]. Whereas electron density was observed only for Nrf2 residues 24–29, the structure revealed a β-turn conformation similar to that of the ETGE motif, as well as a similar peptide interaction mode. A further costructure was subsequently solved at 1.6- Å resolution using a larger DLG peptide incorporating Nrf2 residues 17–51 (Fig. 2B) [39]. Importantly, this structure revealed a more extended and distinct binding interface, as well as an altered Nrf2 peptide conformation consisting of an N-terminal helix (Leu19–Arg25) and two short 3_10_ helices (Ile28 to Leu30 and Arg34 to Phe37). In this revised structure, the DLG motif residues Asp29–Leu30–Gly31 are located similar to the ETGE motif residues Glu79–Thr80–Gly81.

Comparison of the DLG and ETGE peptide complexes shows that they insert in a manner similar to the bottom of the Kelch domain pocket, but that they deviate at their N-terminus owing to the helical conformation of the DLG peptide motif (Fig. 2C). Thermodynamic analyses using isothermal titration calorimetry showed that the Keap1–ETGE interaction was enthalpy-driven, consistent with the large number of electrostatic interactions observed. By contrast, the weaker Keap1–DLG interaction was characterized by a reduced enthalpy, but a small favorable entropy [39]. Differences were also observed in the binding kinetics. The ETGE peptide exhibited a slow on and slow off rate yielding a high-affinity interaction, whereas the weaker binding DLG peptide showed fast kinetics for both its association and its dissociation [39].

These differences are thought to be important for fine-tuning regulation of the stress response [45]. The separate DLG and ETGE motifs allow a single Nrf2 molecule to bind to the two Kelch domains present in the Keap1 dimer. A hinge and latch mechanism has been proposed in which the high-affinity ETGE motif acts as a hinge anchored to the first Kelch domain, whereas the weaker DLG motif is engaged as the latch to maintain minimal housekeeping expression levels of the Nrf2 protein [45]. A model for the full Keap1 E3 ligase complex is discussed further below. Its proper assembly is thought to be important to correctly position the Neh2 domain for ubiquitination and may be disrupted under conditions of cellular stress by modifications to the reactive cysteine residues in Keap1 [24], [38], [44].

The Kelch domain structures also help to explain the effects of somatic cancer mutations identified in Keap1 (Figs. 2A and 2B), as well as those in Nrf2, which cluster to the DLG and ETGE motifs [37], [39], [46]. These mutations destabilize the Keap1–Nrf2 interaction leading to Nrf2 protein stabilization and induced transcriptional responses that protect cancer cells from environmental stresses, including the potential toxicity of cancer drugs or radiation therapy [47], [48], [49].

## 3 Structural basis of Keap1 dimerization

The BTB domain is a protein–protein interaction domain found throughout eukaryotes as well as in the poxvirus zinc finger (POZ) protein family and is consequently sometimes referred to as the BTB/POZ domain [34], [50], [51], [52]. Over 350 different BTB-containing proteins are identified in humans, of which the majority are found in combination with other domains. The BTB fold was first defined by the structure of the promyelocytic leukemia zinc finger (PLZF) protein, which belongs to the human BTB-ZF class of transcriptional repressors [53].

**Fig. 3:**
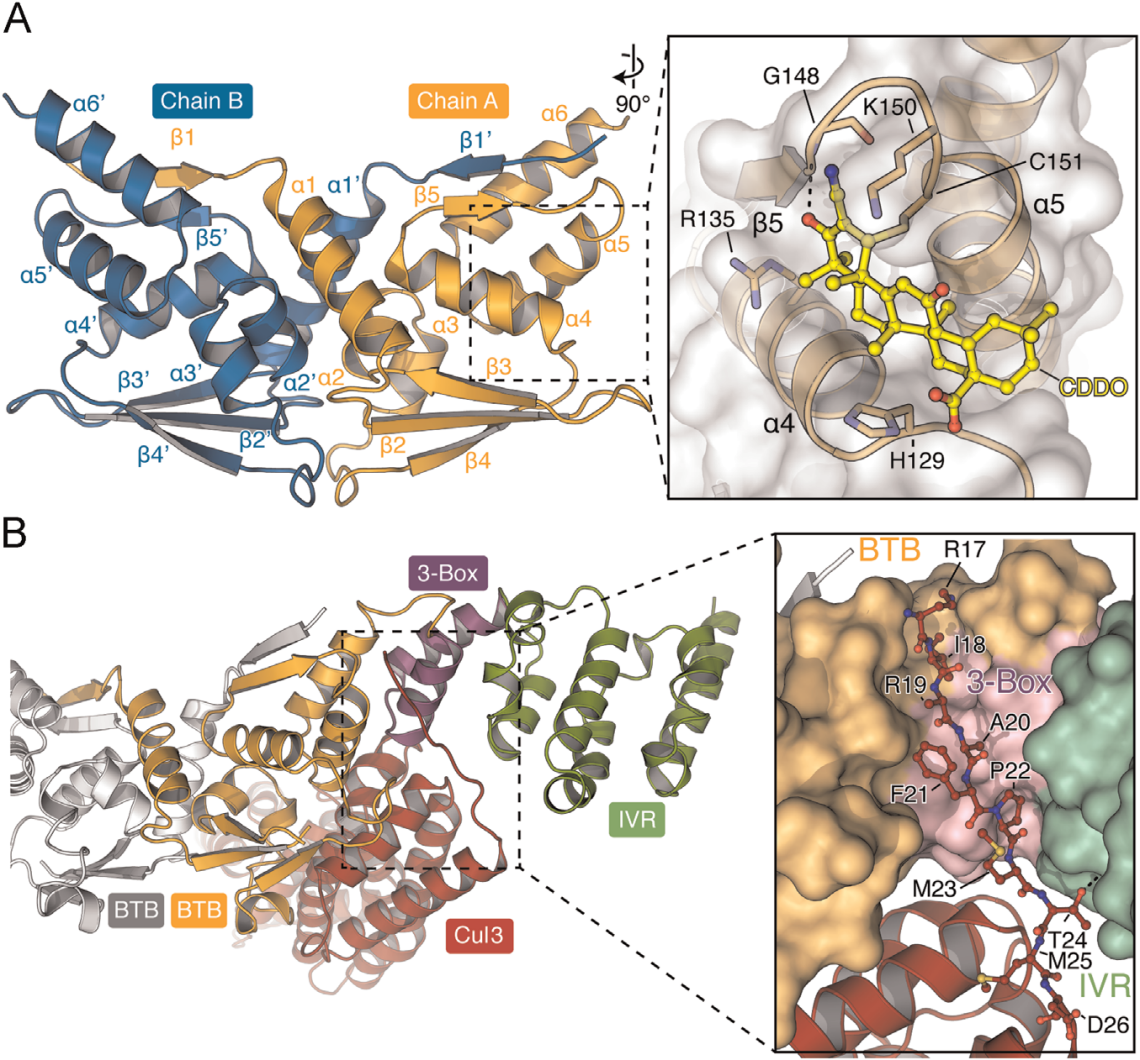
BTB dimerization and Cul3 binding. (A) Ribbon representation of the Keap1 BTB dimer (PDB 4CXI). The inset shows the binding mode of the antagonist bardoxolone/CDDO (PDB 4CXT), which forms a covalent bond to the side chain of Cys151. (B) Model of the Cul3 interface, based on the crystal structure of the KLHL11–Cul3 complex (PDB 4AP2). Cul3 is shown in red, whereas the BTB, 3-box, and IVR domains are colored as shown in Fig. 1. The second BTB subunit is colored gray. The inset shows the interaction between the residues of the Cul3 N-terminal extension and the 3-box of KLHL11.

The structure of the BTB domain of Keap1 was reported in 2014 and shows a fold similar to the PLZF domain despite its low sequence identity and divergent cellular function [35]. Both structures show a tightly intertwined homodimer, with the individual domains related by a crystallographic twofold axis (Fig. 3A). The shared fold comprises a three-stranded β-sheet flanked by six α-helices. Structural elements across the fold contribute to the dimer interface, providing a significant buried surface area in Keap1 of 2001 Å^2^. Notable among these are the long α1 helix and the N-terminal β1 strand, which forms a domain-swapped antiparallel β-sheet with the β5 strand of the other bound subunit. Some members of the BTB-Kelch family have been reported to heterodimerize, such as the KLHL9 and KLHL13 protein pair [54]. However, these two proteins are highly conserved in their primary sequences. The Keap1 protein has no such closely conserved homologs within this family and its BTB domain is thought to exist as an obligate homodimer.

## 4 Structural basis of Keap1 assembly with Cul3

The E3 ligase activity of Keap1 requires its assembly with Cul3 to become part of a larger CRL3 complex [13], [55]. Whereas no structures currently exist for this complex, the structure of the relevant Cul3 N-terminal domain has been solved in complexes with other homologous BTB-Kelch family members, including KLHL3 and KLHL11 [32], [56]. The Cul3 protein forms a stalk-like scaffold that binds and orients the substrate-binding E3 subunit at its N-terminus and a RING protein at its C-terminus [57], [58], [59].

The Cul3 N-terminal domain contains three Cullin repeats, each folded as a five-helix bundle, as well as an N-terminal extension of some 24 amino acids [32]. Each subunit in the BTB dimer binds one Cul3 molecule yielding a large heterotetramer with a twofold symmetry axis across the BTB dimer. The BTB domain is bound primarily by the H2 and H5 helices of the first Cullin repeat, whereas the Cul3 N-terminal extension inserts into an adjacent hydrophobic groove formed by the proximal 3-box motif (Fig. 3B) [32]. The 3-box is a conserved C-terminal extension of the BTB domain found in most Cul3-dependent E3 ligases, including the BTB-Kelch proteins and the MATH-BTB family [58]. Structures including this domain have been solved for the MATH-BTB protein SPOP [58], [60], as well as the BTB-Kelch proteins KLHL3, KLHL11, Gigaxonin (or KLHL16), and KBTBD4 [32], [56], [58]. These show the 3-box motif to consist of two helices (α7 and α8) that pack in an antiparallel four-helix bundle configuration with the two C-terminal helices of the BTB domain (α5 and α6).

The 3-box motif connects the BTB domain to the central portion of the Keap1 protein known either as the IVR domain or the BACK (BTB and C-terminal Kelch) domain [61]. This region was first fully characterized in the KLHL11 crystal structure and contains a further six helices (α9–α14) that pack perpendicular to the 3-box (Fig. 3B) [32]. Bioinformatic analyses suggest that this region is structurally conserved in Keap1 [32], [56], [61].

## 5 Model for Nrf2 degradation by Keap1

A model of the full-length Keap1 homodimer has been obtained at low resolution by single-particle electron microscopy [44]. Reconstruction at 24-Å resolution has revealed two large spheres attached by short linker arms to the sides of a small forked-stem structure. The stem matches the shape and size of the BTB dimer, whereas each sphere would appear to incorporate the Kelch domain positioned atop the IVR domain [44]. This structure provides support for the two-site binding model of Nrf2 in which the Neh2 domain spans the gap between the two Kelch domains, which separately engage the ETGE and DLG motifs. The region between these two degrons has predicted helical structure and contains seven lysine residues as potential ubiquitin-acceptor sites, suggesting that the dimeric Keap1 structure is required to immobilize this Nrf2 segment for efficient ubiquitination.

**Fig. 4:**
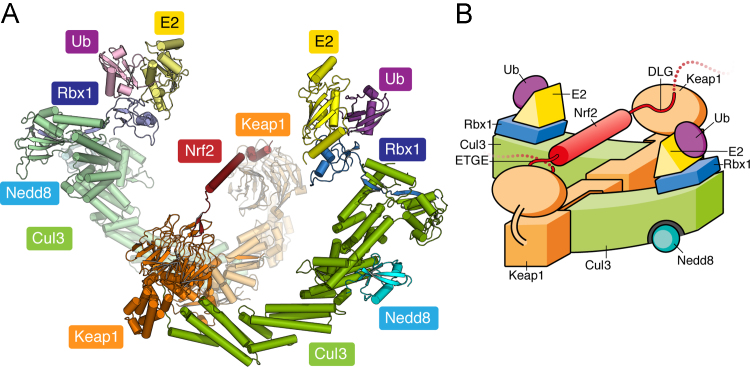
Model of the fully assembled CRL3 complex. (A) Structural model assembled as previously described [32]. A predicted helix in the Neh2 region of Nrf2 is modeled between the bound DLG and ETGE sites. (B) Schematic illustration of the complex shown in (A).

A more complete model of the full CRL3 complex (Fig. 4) has been proposed based on the related KLHL11–Cul3 structure [32] and other known Cul1 [59] and Cul5 [62] complex structures. Such modeling is possible owing to the core elements of the BTB fold that are conserved in the structures of the Cul1-adaptor protein Skp1 [63] and the Cul2/5-adaptor ElonginC [64], [65], [66], [67]. These adaptors bind to different E3 ligases containing F-box and SOCS box motifs, which are functionally analogous to the 3-box, although distinct in structure. The final model includes the Cul3 C-terminal domain as well as the bound RING domain protein Rbx1, which recruits the charged E2-ubiquitin moiety. Neddylation (the covalent attachment of the small protein Nedd8) to a specific lysine in the Cul3 C-terminal domain is thought to modulate the conformation of this complex allowing the closest association of the substrate and E2-ubiquitin [62]. Significantly, the model places the E2-ubiquitin subunits centrally and above the Neh2 domain, where they seem poised to participate in Nrf2 ubiquitination (Fig. 4). The precise mechanism of polyubiquitination remains to be elucidated.

## 6 Structural insights into Keap1 inhibition

Chemical inducers of Nrf2 that block Keap1 function have proven an effective mechanism to exploit the antioxidant response in the fight against human disease. Indeed, the Nrf2 inducer BG-12, a formulation including dimethyl fumarate, was recently approved for the treatment of relapsed multiple sclerosis [68]. The majority of antagonists are electrophiles that covalently modify the free cysteines in the Keap1 protein. The mechanism of action of one such molecule, the triterpenoid bardoxolone (also known as CDDO), has been revealed by a cocrystal structure with the BTB domain of Keap1 [35]. This molecule binds covalently to Keap1 Cys151 and nestles in a shallow groove between the α4 and the α5 helices (Fig. 3A). Although the interaction induces subtle rearrangements in the local vicinity of Keap1, the bulk of bardoxolone is thought to inhibit Cul3 binding by blocking the adjacent hydrophobic groove of the 3-box [35]. This loss of binding prevents the recruitment of E2-ubiquitin and abrogates Nrf2 ubiquitination.

Other electrophiles are expected to bind to Cys273 and Cys288 in the IVR domain [17]. These molecules are predicted to distort the IVR domain fold. Any such perturbation could alter the stable positioning of the associated BTB and Kelch domains and so induce an improper geometry for ubiquitination or, alternatively, affect protein–protein interactions in the Cullin–RING ligase complex [32], [40], [56]. Severe perturbations might be expected to break the tethering of Nrf2, consistent with the dissociation observed in studies of the mouse Keap1 protein [18]. However, ITC experiments using chemical modifiers of human Keap1 have suggested that the overall affinity for binding of the Neh2 domain is unchanged [40]. Thus, it is likely that the integrity of the Kelch domain fold is not affected. Without productive ubiquitination, the stable association of Nrf2 with Keap1 is expected to block further substrate binding and turnover, leading to the accumulation of newly synthesized Nrf2 and the consequent induction of the antioxidant response [69]. However, a precise understanding of the consequences of these electrophiles awaits additional structural studies.

More recently, a number of cocrystal structures have been reported for small-molecule inhibitors that target the Kelch domain noncovalently and compete directly for binding with the Nrf2 peptide [70], [71]. These molecules occupy the same shallow pocket in Keap1 used by the ETGE and DLG motifs (Fig. 2D). This pocket has a markedly electropositive surface potential and is rich in basic arginine side chains. As a consequence, the most potent binders contain acidic groups that are challenging for cell permeability. Nonetheless, the ability of small molecules to target the BTB, the IVR, or the Kelch domain presents a welcome toolbox for biologists and a diverse set of therapeutic strategies for evaluation in preclinical and clinical studies [23].

## 7 Conclusions

A combination of biochemical and structural studies has yielded remarkable insights into the molecular mechanisms controlling the cellular response to oxidative and electrophilic stress. Importantly, the structural models are consistent with the proposed two-site hinge and latch model for Nrf2 capture and reveal sensible spatial arrangements for its subsequent ubiquitination. Their construction is particularly enabled by the modular nature of the Cullin–RING ligases, which allows different structures to be assembled together for an understanding of the entire complex. However, the lack of a high-resolution full-length Keap1 structure currently limits our knowledge in several respects. Progress here is perhaps hindered by the short linker that connects the IVR and Kelch domains. For example, the structure of the MATH-BTB protein SPOP shows an analogous linker that is highly flexible leading to asymmetry in the dimeric protein, as well as the E3-substrate complex [58]. Thus, it remains an open question whether rigidity or partial flexibility is optimal for Keap1 activity. Uncertainty about the position of the Kelch domain, as well as the undetermined structure of the Keap1 IVR domain, also means that there is still much to learn about the cysteine code.

Overall, the lessons learned from the Keap1–Nrf2 system provide a prototype model for our understanding of the entire BTB-Kelch family. Structural studies across this family are less advanced, but already reveal a diversity in the Kelch domain substrate pocket, as well as alternative peptide binding conformations and interfaces [32], [72], [73]. These data support the general concept of targeting the Kelch domain with small molecules to selectively interfere with substrate recruitment, whereas the cysteine-rich sensor mechanism currently appears unique to Keap1.
